# Hybrid neural network models for time series disease prediction confronted by spatiotemporal dependencies

**DOI:** 10.1016/j.mex.2024.103093

**Published:** 2024-12-09

**Authors:** Hamed Bin Furkan, Nabila Ayman, Md. Jamal Uddin

**Affiliations:** a Department of Statistics, Shahjalal University of Science and Technology, Sylhet, Bangladesh; b Department of Computer Science & Engineering, University of Chittagong, Chittagong, Bangladesh; c Department of General Educational and Development, Daffodil International University, Dhaka 1216, Bangladesh

**Keywords:** Time series prediction, Disease outbreak prediction, Disease forecast, Influenza outbreaks, Hybrid neural network, Neural network comparison, Hybrid Neural Networks

## Abstract

In infectious disease outbreak modeling, there remains a gap in addressing spatiotemporal challenges present in established models. This study addresses this gap by evaluating four established hybrid neural network models for predicting influenza outbreaks. These models were analyzed by employing time series data from eight different countries to challenge the models with imposed spatial difficulties, in a month-on-month structure. The models' predictions were compared using MAPE, and RMSE, as well as graphical representations generated by employed models. The SARIMA-LSTM model excelled in achieving the lowest average RMSE score of 66.93 as well as reporting the lowest RMSE score for three out of eight countries studied. In this case also, GA-ConvLSTM-CNN model comes in second place with an average RMSE score of 68.46. Considering these results and the ability to follow the seasonal trends of the actual values, this study suggests the SARIMA-LSTM model to be more robust to spatiotemporal challenges compared with the other models under examination.

This study

• Evaluated established methods with unique imposed difficulty.
• Addressed spatiotemporal characteristics of the data.
• Proposed the SARIMA-LSTM model based on evaluation metrics.

## Graphical abstract

**Figure:**
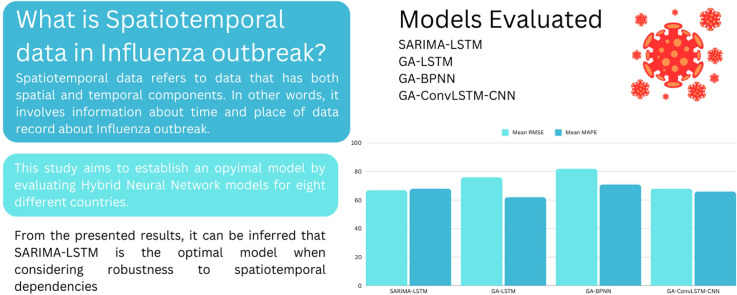

Specifications table

**Table:** 

Subject area:	Bioinformatics
More specific subject area:	Disease outbreak prediction in spatiotemporal context
Name of your method:	Hybrid Neural Networks
Name and reference of original method:	Kara A. Multi-step influenza outbreak forecasting using deep LSTM network and genetic algorithm. Expert Syst Appl. 2021 Oct 15;180:115153
Resource availability:	https://github.com/07hamed/IDO-hybrid_ml_models

## Background

Infectious disease modeling is a field of study that involves the use of statistical and computational models to simulate and analyze the spread of diseases within a population in a specific time interval. Influenza is a significant infectious disease that leads to substantial setbacks in public health. Its incidence often ranks at the top among notifiable infectious diseases [1]. It is an acute respiratory infectious disease caused by the influenza virus, posing a serious threat to human health [2]. Worldwide, influenza is estimated to result in approximately 3 to 5 million annual cases of severe illness and approximately 250,000 to 500,000 deaths [3]. Timely and accurate prediction of the outbreak of this type of disease can aid the authority in making interventions and prevent the mass loss of resources and lives.

However, the effectiveness of predictive models in this context is intricately tied to the high sensitivity of spatiotemporal characteristics [4]. This study aimed to provide a deeper understanding and evaluation of the performance of predictive models in a spatiotemporal Influenza outbreak context.

Therefore, reviewing extensive literature on predictive modeling for influenza outbreaks, the authors of this study identified two main approaches to model the disease outbreak: i) Pure models, which come in two types: a) Traditional statistical models; b) Machine learning and neural network models and ii) Hybrid neural network models. The second approach to disease outbreak prediction, hybrid neural network models, is gaining popularity [5] due to their flexible structure and the accuracy they provide. Disease outbreak data are generally multivariate in nature, having spatial-temporal dependence, and dynamic and nonlinear characteristics. While classical pure models are prone to difficulties like non-feasible assumptions [6], overfitting and underfitting [7,8], multivariate high dimensional data and spatiotemporal dependency characteristics [9], hybrid neural network models can learn complex patterns and deep insights hidden in the data while keeping the flexibility and robustness intact as depicted in the already published scientific studies. However, to the best of our knowledge, there is a significant gap in the scientific literature: there are no studies that systematically assess established hybrid models in spatiotemporal contexts.

The significance of this study lies in its aim to identify a model with minimal spatial dependency that excels in predicting temporal progression. This model could be instrumental in monitoring the evolution of influenza outbreaks at regional or sub-continental levels, an area which has not been explored to date.

In pursuit of this objective, the study seeks to evaluate the performance of established hybrid neural network models in eight different countries to predict influenza outbreaks while ensuring robustness to spatiotemporal differences. However, it is crucial to understand that this study does not intend to achieve the optimum results in prediction, but to evaluate the prediction's robustness. As depicted in the noble studies, statistical and machine learning pure models are outperformed by hybrid neural network models, this study decided to evaluate only the hybrid models listed below:

i. SARIMA-LSTM – [10]
ii. GA-LSTM - [11]
iii. GA-BPNN – [12]
iv. GA-ConvLSTM-CNN - [13].

Authors have employed the Influenza outbreak data of 8 different countries Afghanistan, Bangladesh, Bhutan, India, Maldives, Nepal, Pakistan, and Sri Lanka from the southeast Asia region over 15 years monthly. These countries have been selected based on their similarities in geographical and socio-economic conditions. The influenza outbreak data were collected from Our World in Data [Originally from WHO sources], and data on environmental variables are collected from Open Meteo.

## Method details

### Data collection and processing

The response variable of this study; confirmed cases of Influenza for all strains, was collected from Our World in Data [14], primarily provided by the World Health Organization (WHO) at FluNet [15]. This data is available on a weekly and monthly basis. Most of the literature on Influenza outbreaks has used the data in weekly format, however, this study considered the monthly data because of evident excessive zero (0) values for most of the countries in weekly format due to the smaller population of the respective countries.

Climatological factors like Relative humidity (2m), Temperature (2m), Precipitation (Snow + Rain), and Solar Radiation were collected from Open-Meteo [16], as [17] depicted the different degree of influence of climatological factors on Influenza outbreak. These data were provided on an hourly basis and needed preprocessing by averaging throughout the day and grouped by month of the year to get them on the desired monthly basis to align them with the response variable.

All the data processing and model structure for the predictive models were coded in Python language on Google Collaboratory [18]. It incorporates data from January 2009 to June 2023 on a month-on-month basis and aims to evaluate the model's performance by making predictions for the latest 12 months. This study relies on the original studies only for information provided by the respective authors to reproduce the models. Consequently, the results of this study are expected to differ from theirs.

### Structure of the established hybrid models

#### SARIMA-LSTM

**Fig. 1:**
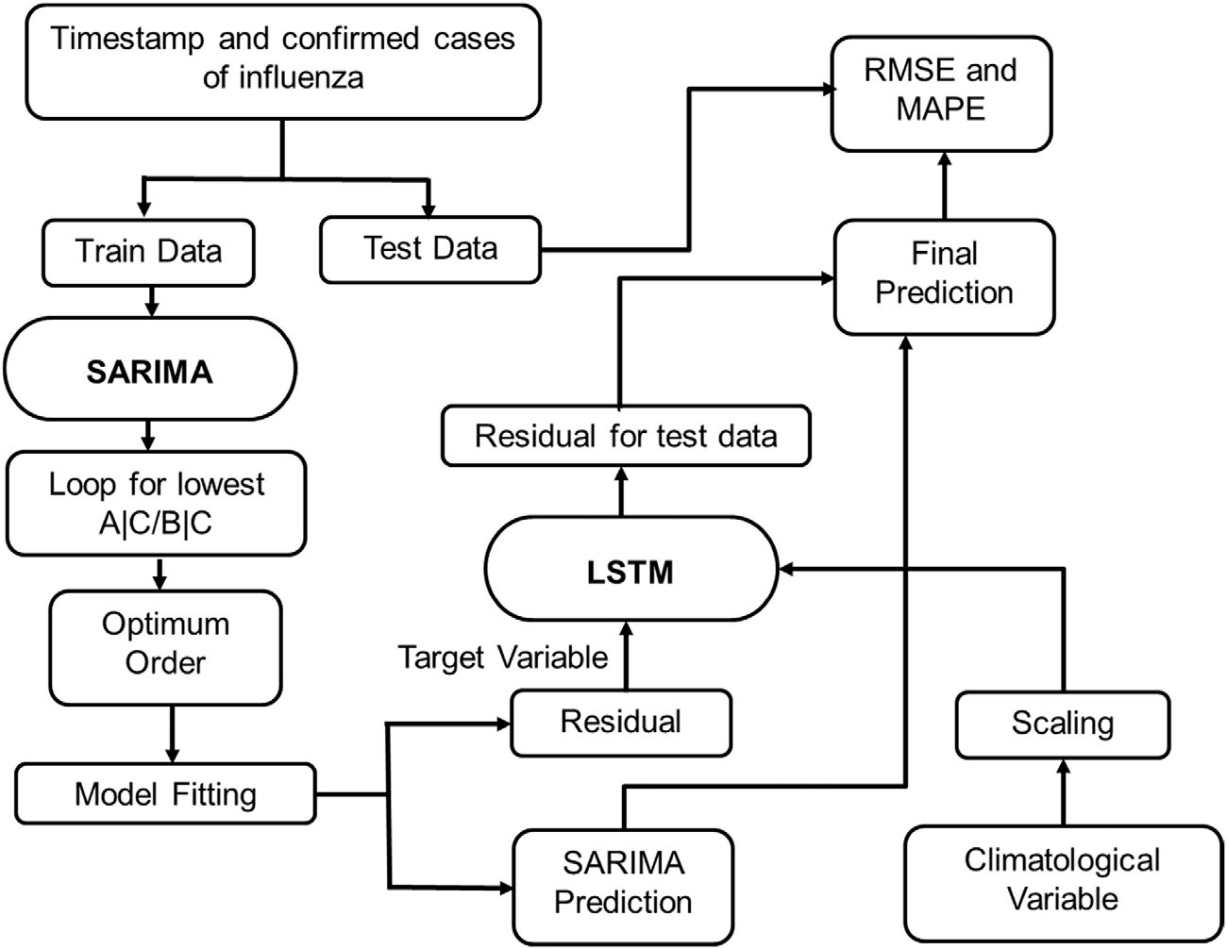
Methodology of SARIMA-LSTM hybrid model.

[19] proposed the implementation of the SARIMA-LSTM hybrid structure to combine these two components. They employed a two-step traditional ensemble technique to concatenate these two models by applying and fitting the SARIMA model, extracting the residuals, and feeding the residuals to the LSTM model to predict residuals, to curve out the nonlinear complexities unexplained by the SARIMA model [Fig. 1]. This study found optimal SARIMA orders for different countries as Afghanistan (1, 1, 1), (0, 1, 1, 12), Bangladesh (1, 1, 1), (0, 1, 2, 12), Bhutan (3, 1, 4), (0, 1, 2, 12), India (1, 1, 2), (1, 1, 1, 12), Maldives (1, 1, 1), (0, 1, 1, 12), Nepal (2, 1, 1), (0, 1, 1, 12), Pakistan (2, 1, 3), (1, 1, 2, 12), and Sri Lanka (1, 1, 2), (0, 1, 1, 12). These orders were obtained by evaluating AIC and BIC scores. Then the SARIMA model was fitted to data using these orders and predictions for the last 12 months from July 2022 to June 2023 were recorded. Residuals from this SARIMA model conjugated with the LSTM network provided the final predictions for the SARIMA-LSTM model.

#### GA-LSTM

**Fig. 2:**
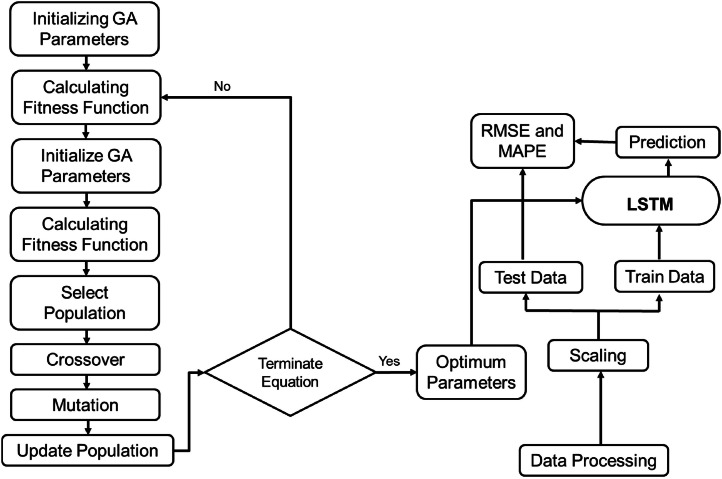
Methodology of GA-LSTM hybrid model.

This hybrid structure is proposed by [20], and a streamlined process of optimizing LSTM is also suggested in the article that was employed in this study. However, the GA parameters were optimized to better fit the different structures of the data employed by this study. The data were fed into the GA function with Adam optimizer connected with an LSTM model that returned the optimal parameter combination for Afghanistan (1, 22, 41), Bangladesh (4, 52, 115), Bhutan (11, 32, 96), India (14, 6, 119), Maldives (1, 60, 72), Nepal (1, 57, 28), Pakistan (15, 54, 35), and Sri Lanka (11, 17, 19) for the search of window size (w), the unit size (u), and the number of epochs (e) parameters based on validation RMSE [Fig. 2]. Then the LSTM model for the respective countries was built using these parameter combinations.

#### GA-BPNN

**Fig. 3:**
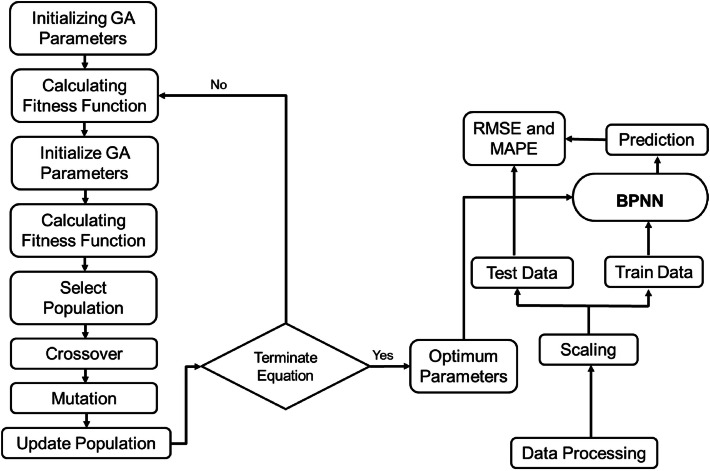
Methodology of GA-BPNN hybrid model.

Although the system solely based on the BPNN method provides sound results, [21,22] have shown BPNN method conjugated with optimization and search algorithms like genetic algorithm and evolution algorithm makes the model more stable [23]. In this study, the GA algorithm is employed to obtain the best value of the parameter's best learning rate from 0.001 to 0.1, the best number of neurons from 8 to 128 neurons, and the best activation from the array of linear, relu, sigmoid, and tanh functions based on validation RMSE [Fig. 3].

#### GA-ConvLSTM-CNN

**Fig. 4:**
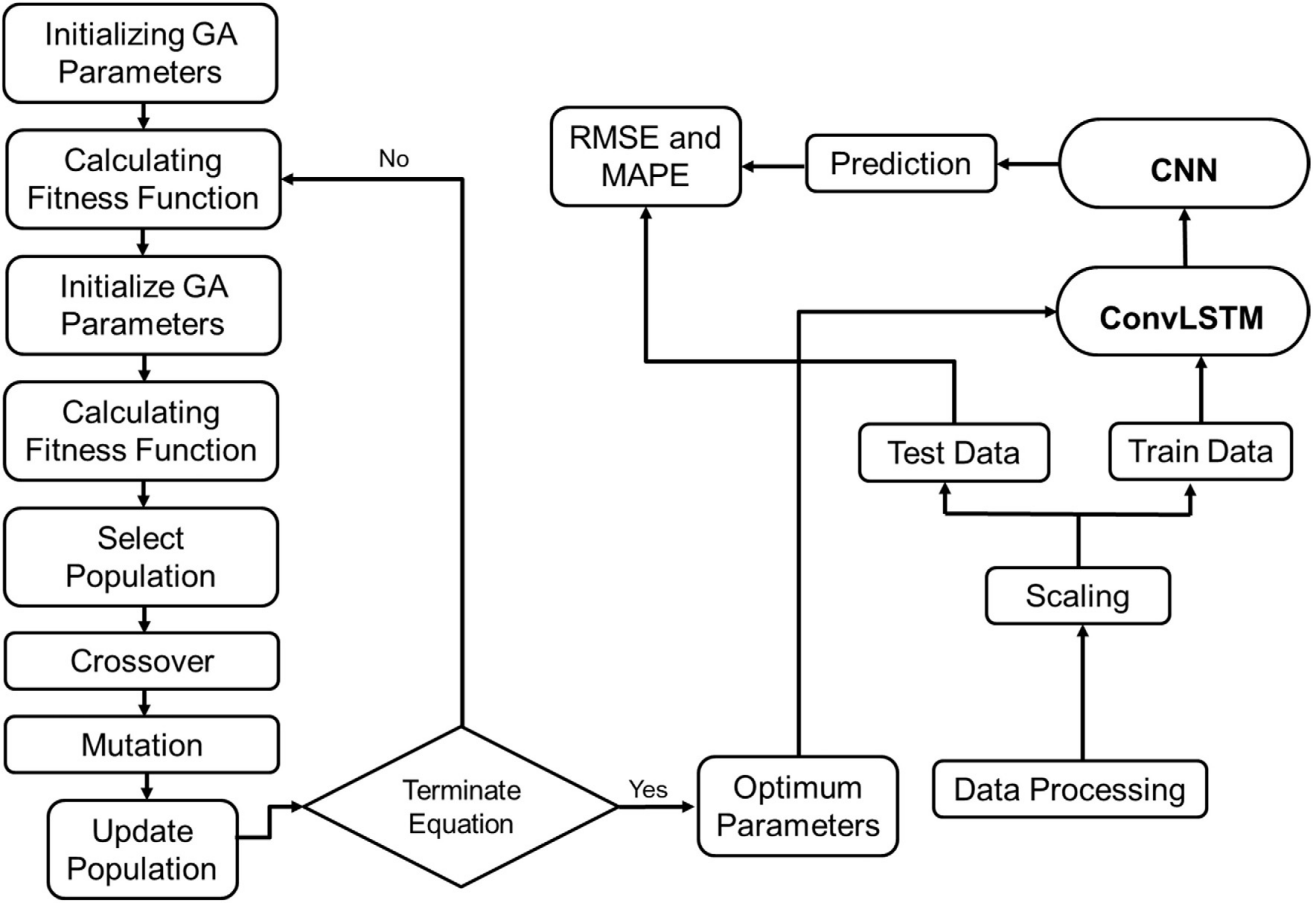
Methodology of GA-ConvLSTM-CNN hybrid model.

This structure contained a GA algorithm on top of two fully connected ConvLSTM layers and two fully connected CNN layers [24] to obtain optimal values for window size (w), the number of units (u), and the number of epochs (e) for countries as Afghanistan (1. 2, 113), Bangladesh (2, 24, 122), Bhutan (15, 24, 64), India (2, 24, 89), Maldives (14, 4, 120), Nepal (1, 57, 28), Pakistan (12, 24, 82), and Sri Lanka (9, 36, 81) [Fig. 4]. These values were obtained based on the lowest validation RMSE for training data and implemented on the hybrid model for test data.

### Evaluation metrics

[25], suggested Root Mean Squared Error (RMSE), and Mean Absolute Percentage Error (MAPE). MAPE expresses the absolute deviation of the predicted value from the actual values in percentage notation. It is robust from the different order magnitude of different spatiotemporal settings [26] hence, in the context of this study, making it more appropriate to evaluate the performance of individual models for different countries. The MAPE is calculated as

$$\text{Mean}\;\text{Absolute}\;\text{Percentage}\;\text{Error}\;\left(\text{MAPE}\right)\;=\frac{1}{n}*\Sigma \frac{\left(\left|Actual-Predicted\right|\right)}{Actual)}*100$$

The equation for RMSE is,

$$\text{Root}\;\text{Mean}\;\text{Squared}\;\text{Error}\;\left(\text{RMSE}\right)\;=\surd \left[\frac{\sum {\left(Actual-Predicted\right)}^{2}}{n}\right]$$

The visual representations of the predictions made by the different models for each country are depicted by the line chart of true values vs. predicted values from each model. This line chart is generated using Python's data visualization library namely Matplotlib.

## Method validation

**Fig. 5:**
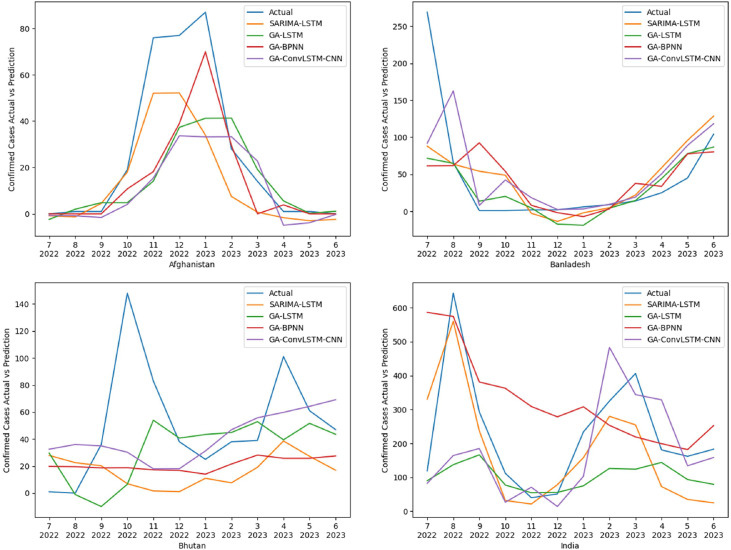
Comparative performance of models using the data of countries: Afghanistan, Bangladesh, Bhutan, and India.

**Fig. 6:**
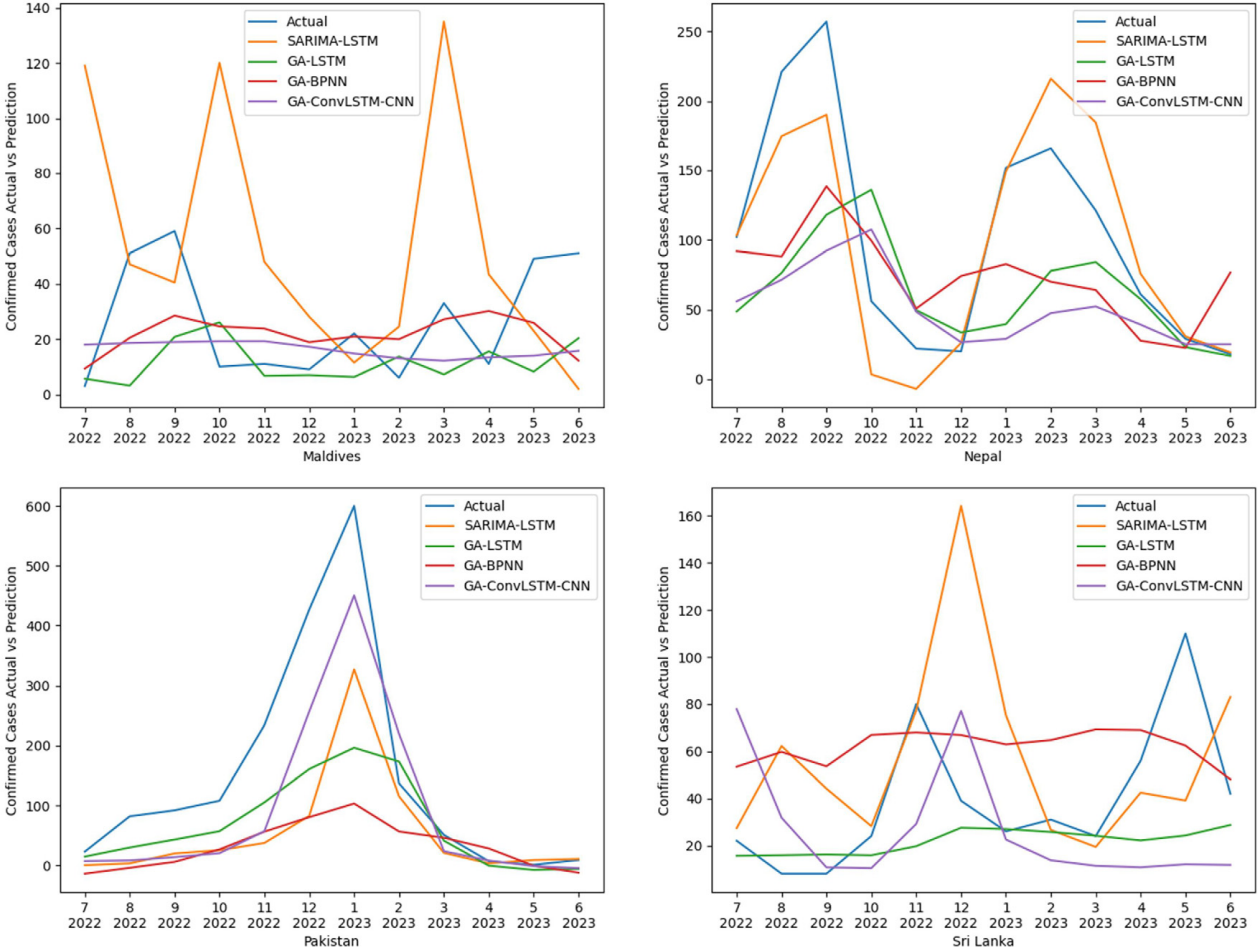
Comparative performance of models using the data of countries: Maldives, Nepal, Pakistan, and Sri Lanka.

The comparative visual of the performance of these models are presented in line charts over time [Fig. 5], [Fig. 6] where the confirmed case of Influenza is plotted on the y-axis and respective month and year is plotted on the x-axis. The eight countries were divided into two figures with subplots. In [Fig. 5] are the performance for Afghanistan, Bangladesh, Bhutan, and India.

The visual representation for the models of Maldives, Nepal, Pakistan, and Sri Lanka are presented in [Fig. 6]

[Table 1] gives information about the MAPE scores of different models for the respective countries, the MAPE scores for various models in different countries show that each new model has excelled in two instances. Based on the average MAPE score for these models, the GA-LSTM model has the superiority with a score of 62.38%. And the model GA-BPNN has the largest average MAPE with a score of 71.21%. Considering all the instances, it is recorded that, the SARIMA-LSTM model has the most extreme deviation of 112.42% in the case of Maldives, and this model produced the lowest MAPE score across all the instances for Nepal, with a score of 27.43%.

**Table 1:** Mean Absolute Percentage Error.

	SARIMA-LSTM	GA-LSTM	GA-BPNN	GA-ConvLSTM-CNN
Afghanistan	49.96%	61.40%	46.74%	66.52%
Bangladesh	79.67%	64.63%	87.10%	79.23%
Bhutan	83.33%	61.14%	71.32%	59.91%
India	43.55%	68.57%	65.89%	48.29%
Maldives	112.42%	74.6%	65.57%	78.55%
Nepal	27.43%	56.74%	57.86%	64.31%
Pakistan	64.09%	53.51%	81.33%	49.63%
Sri Lanka	87.60%	52.5%	83.94%	83.41%
Average	68.38%	62.38%	71.21%	66.23%

From [Table 2] it is evident that SARIMA-LSTM has the advantage over other models referring to the lowest average RMSE of 66.93 and also achieving the lowest RMSE in three instances. GA-LSTM and GA-ConvLSTM-CNN models have excelled for two countries each while the GA-BPNN model achieved the lowest RMSE score in only one case. GA-BPNN has the largest average RMSE score of 81.69 among the other models.

**Table 2:** Root Mean Squared Error.

	SARIMA-LSTM	GA-LSTM	GA-BPNN	GA-ConvLSTM-CNN
Afghanistan	19.68	25.27	21.13	27.15
Bangladesh	59.58	59.34	68.8	61.65
Bhutan	55.75	48.07	50.24	44.12
India	111.29	211	197.67	163.52
Maldives	56.61	25.03	20.44	26.47
Nepal	37.66	76.60	70.32	86.34
Pakistan	144.89	128	188.93	95.66
Sri Lanka	49.44	33.17	36	41.80
Average	66.93	75.81	81.69	68.46

Regarding the problem of predicting influenza cases, the performance of hybrid models tends to vary according to the temporal features provided as different models seem to achieve best performance while predicting influenza cases of individual country.

From the presented results, it can be inferred that SARIMA-LSTM is the optimal model when considering robustness to spatiotemporal dependencies, it showed prowess in capturing the trend of actual values in all the eight different countries and reporting the lowest RMSE score for three countries Afghanistan, India, and Nepal. In the cases of India, and Nepal it provided extremely good predictions reflected by the RMSE and MAPE difference from the other models. Regarding the average RMSE score, SARIMA-LSTM is the best-performing model and measured on the MAPE score, it comes in the 3rd position. This model deviated the most in the case of Maldives with a 112.42% MAPE score but also generated the lowest MAPE score among all the combinations of 27.43% in the case of Nepal.

The GA-LSTM model has performed consistently in all eight countries with the best average MAPE score of 62.38% for all the countries ranging from 52.5% to 74.6%. The average RMSE score for this model is 75.81, which comes in the 3rd position. The lowest average MAPE score reflects its ability to replicate the trends in the actual values, however the line chart for GA-LSTM shows, that it takes a more generalized approach compared to the SARIMA-LSTM model in capturing the trends resulting in greater RMSE than the SARIMA-LSTM model but a lower MAPE. The consistent results without the presence of extreme values make it the most robust model among all the employed models.

The GA-BPNN models come in the last position in terms of both RMSE and MAPE scores at 81.69 and 71.21% respectively. Also, from [Fig. 5], and [Fig. 6] the deviation from the actual values is visible, as it failed to capture the seasonal trend in most of the cases, but it managed to achieve the lowest MAPE score for Afghanistan and Maldives. Considering the RMSE scores, it only managed to achieve the best results for Maldives.

In contrast, the GA-ConvLSTM-CNN model, with an average MAPE score of 66.23% and an average RMSE score of 68.46, emerges as a noteworthy contender among the models evaluated. While it may not have secured the top position in the overall rankings, its performance underscores its stability for spatiotemporal contexts. For most of the countries, it was able to replicate the seasonal trends, excelling in the cases of Bhutan and Pakistan, this model came in 2nd position for both metrics considered.

Considering the presented discussion and data from Table 3, each model is assessed for its distinct strengths and weaknesses. The SARIMA-LSTM model demonstrates proficiency in capturing both seasonality and trends in the data but occasionally tends to overestimate predictions. On the other hand, the GA-LSTM model excels at trend capture but exhibits reluctance in considering extreme values. The GA-BPNN model, while occasionally responsive to extreme values, is somewhat hesitant in capturing seasonality patterns and often generalizes its predictions. Lastly, the GA-ConvLSTM-CNN model exhibits responsiveness to extreme values but occasionally underestimates predictions.

**Table 3:** Model comparison and contrast.

Models	Mean RMSE	Mean MAPE	Strength	Weakness
SARIMA-LSTM	66.93	68.38%	Seasonality, Trend capturing	Occasional overestimation
GA-LSTM	75.81	62.38%	Trend capturing	Reluctant to extreme values
GA-BPNN	81.69	71.21%	Occasionally responsive to extreme values	Reluctant to seasonality
GA-ConvLSTM-CNN	68.46	66.23%	Responsive to extreme values	Occasional underestimation

## Limitations

Not applicable.

## Ethics statements

This research does not involve human or animal subjects and no data were collected from social media platforms. The data source has been cited in the article and its bibliographic reference is available in Reference section.

## CRediT authorship contribution statement

**Hamed Bin Furkan:** Conceptualization, Methodology, Investigation, Data curation, Writing – original draft, Visualization, Writing – review & editing. **Nabila Ayman:** Methodology, Software, Data curation, Writing – original draft, Visualization. **Md. Jamal Uddin:** Supervision, Validation, Writing – review & editing.

## Declaration of competing interest

The authors declare that they have no known competing financial interests or personal relationships that could have appeared to influence the work reported in this paper.

## Data Availability

Data will be made available on request.
